# Prevalence and health correlates of Onine Fatigue: A cross-sectional study on the Italian academic community during the COVID-19 pandemic

**DOI:** 10.1371/journal.pone.0255181

**Published:** 2021-10-14

**Authors:** Andrea Bonanomi, Federica Facchin, Serena Barello, Daniela Villani

**Affiliations:** 1 Department of Statistical Science, Università Cattolica del Sacro Cuore, Milan, Italy; 2 Department of Psychology, Università Cattolica del Sacro Cuore, Milan, Italy; 3 EngageMinds HUB–Consumer, Food & Health Engagement Research Center, Università Cattolica del Sacro Cuore, Milan, Italy; Universitat de Valencia, SPAIN

## Abstract

**Background:**

During the COVID-19 pandemic, many people had to shift their social and work life online. A few researchers and journalists described a new form of fatigue associated with a massive use of technology, including videoconferencing platforms. In this study, this type of fatigue was referred to as Online Fatigue. A new tool (the Online Fatigue Scale) was developed, and its psychometric properties were evaluated. This tool was used to assess Online Fatigue among Italian academics and to examine its associations with psychological and physical health.

**Methods:**

An online survey was conducted in December 2020 on a sample of Italian academics. Besides the Online Fatigue Scale (11 items) used to assess Online Fatigue, the survey was composed of questionnaires (including validated measures) focused on sociodemographic and job-related information, technostress creators, health status, psychological well-being, and COVID-related perceived distress. The psychometric properties of the Online Fatigue Scale were evaluated, and statistical analyses were conducted to examine the associations between Online Fatigue and all the other variables.

**Results:**

Participants were 307 academics aged 24–70 years old (mean age = 40.7; SD = 10.1). The Online Fatigue Scale showed good psychometric properties. Two subscales were identified: Off-Balance Fatigue and Virtual Relations Fatigue. High levels of Off-Balance Fatigue were associated with a greater use of technology, female gender, and presence of minor children. Participants with high scores on both subscales reported a greater frequency of psychosomatic symptoms, unhealthy habits, poorer psychological well-being, and greater Covid-related perceived distress.

**Conclusions:**

The Online Fatigue Scale can be considered a reliable tool to assess Online Fatigue, which was significantly detected in our sample of Italian academics, along with its negative effects on physical and psychological health. Being a woman and having young children represent important risk factors. Universities should promote the separation between work and private life by encouraging self-care activities.

## Introduction

Since the very beginning of its outbreak, the COVID-19 pandemic and the containment measures taken by the governments (including lockdowns) involved shifting our social interactions and work life online [1]. From a public health perspective, physical separation and stay-at home prescriptions are essential to limit the spread of the virus [2], but at the same time these interventions, along with the perceived impact of COVID-19 on people’s daily life and the frequent exposure to distressing news in the media, are associated with anxiety, depression, loneliness, reduced social support, and financial concerns [3–6].

As recently reported by the World Health Organization (WHO), people’s feelings of distress, complacency, alienation, and hopelessness, along with decreased motivation to engage in protective behaviors and information search, represent a normal reaction to the current sustained and unresolved crisis, which has been referred to as *pandemic fatigue* [7]. On the other hand, there is evidence that the intense work-related psychological pressure experienced by frontline healthcare workers during the pandemic may lead to burnout (especially in terms of emotional exhaustion) and somatic symptoms [8]. Moreover, for the whole working population, an additional risk of developing stress and psychological discomfort arises from the negative consequences of being “socially distanced” [9], and sometimes this exacerbated social inequities [10]. Due to the remote working conditions, employees often experienced feeling of loneliness and isolation [11]. Since there were no face-to-face interactions with their boss and colleagues, employees felt frustrated and disappointed and struggles with managing and coordinating work teams [12]. Overall, employees needed to complete their tasks along with the additional responsibilities they have in their house and uncertainties, which have blurred the fine line between work life and personal life [13]. All these risks may then generate additional emotional discomfort, increase work injuries, and even encourage counter-productive behaviors such as absenteeism [14].

Overall, the available body of research on the psychological impact of the pandemic–either on the general population [15, 16] or on specific categories such as healthcare professionals [8]–suggests that fatigue represents not only one of the listed symptoms of COVID-19, but also an important component of people’s subjective experience [17, 18]. In general, fatigue can be an epiphenomenon of a physical disease, a manifestation of an underlying psychological problem, or both. It refers to a condition that ranges from acute to chronic in nature and can result in an overwhelming sense of tiredness, decreased energy, and exhaustion, which may also impair physical and cognitive functions [19].

Since early March 2020, a significant number of mass media outlets (e.g., Forbes, the BBC, National Geographic)–but very few researchers–raised attention to a specific form of pandemic-related fatigue associated with the exponentially increased use of videoconferencing programs as a consequence of remote working [1, 20]. This new COVID-19-related condition has been referred to as *Zoom fatigue* and is characterized by feelings of stress, tiredness, anxiety and concerns, and even burnout due to overusing videoconferencing technology [1]. At the end of 2019, Zoom users were approximately 10 million, while in April 2020 people attending Zoom meetings were 300 million [21]. Considering the variety of the available videoconferencing programs (e.g., Zoom, Skype, Microsoft Teams, Cisco Webex), one can hypothesize that such a new form of pandemic-related fatigue can be widely and intensely experienced by people who work remotely [20].

The biopsychosocial mechanisms associated with the mental and physical toll (e.g., headaches and vision problems) of videoconferencing technology overuse remain unclear. Besides technical malfunctions, it has been suggested that minimal delays occurring in this type of communication, along with lack of mutual gaze, decreased nonverbal cues, and one’s overexposure to one’s own image, cause extra work to the brain to restore synchrony, as well as psychological fatigue [1].

### A new form of pandemic-related fatigue: Online Fatigue

Overall, remote working involves an intense use of internet and technology on a daily basis, and for this reason one can speculate that workers’ experience of fatigue is related not only to videoconferencing, but more generally to the new work modalities imposed by the pandemic. In the current study, this specific type of pandemic-related fatigue, deriving from overusing internet and technology during homeworking, was referred to as Online Fatigue. Thus, Online fatigue can be conceived as a psychological fatigue exacerbated during COVID-19 pandemic that includes both what other authors called Zoom fatigue, such as the feeling of exhaustion associated to the overuse of a particular videoconferencing platform [1, 20], and the stress related to the massive use of videoconferencing activities along with managing private life at home [22, 23].

The existence of a connection between new technologies and stress is well known in psychological literature, as indicated by the notion of *technostress*, i.e., “the stress that users experience as a result of application multitasking, constant connectivity, information overload, frequent system upgrades and consequent uncertainty, continual relearning and consequent job-related insecurities, and technical problems associated with the organizational use of Information and Communications Technology (ICT)” [24]. This definition finds a full correspondence in the classification proposed by Tarafdar et al. [25], who identified five techno-stressors: 1) technological overload, which refers to the feeling of being forced by ICTs to work faster and longer, or to change work habits; (2) techno-invasion, due to the fact that ICTs blur the boundaries between work and private life; (3) techno-complexity, related to feeling inadequate in coping with the complexity of ICTs; (4) technological insecurity, related to the feeling of being threatened about losing one’s job; and (5) technological uncertainty, associated with the need to face continuous updates and to constantly learn new aspects of ICTs. According to a recent systematic review [26], technostress involves a state of psychophysiological activation and negative feelings such as irritability, anxiety and exhaustion, cognitive symptoms such as poor concentration, mental fatigue and memory disturbances [27, 28], but also behavioral strain and sleep problems [29].

In the current pandemic, many workers had to quickly learn to use new remote working tools and platforms, to find creative ways to combine their overlapping multiple roles and to live with the expectation of always being connected, available and operative [22]. Higher education institutions and their employees have not been exempted from this shift to remote work, which involved a remarkable (although understudied) reshaping of academics’ work life, with the consequent revision of educational perspectives and approaches [30–32]. However, very little is known about whether and how academics were affected by the changes in working life due to the pandemic. In this population, remote working entailed a massive use of technologies, which also included multiple types of videoconferencing activities (e.g., online teaching, institutional meetings, research meetings), along with managing family/private life and dealing with the general worries about the pandemic. On the one hand, the academic community has its’ own specificities, but on the other hand the scenario depicted above may reflect the conditions of the increasing number of remote workers in general. For this reason, the academic staff may represent a “prototypical population” to investigate Online Fatigue.

On these premises, we conducted the current study to: (1) provide a preliminary conceptual definition of Online Fatigue, as well as to develop and validate a specific assessment tool (i.e., “Online Fatigue Scale”–see **S1 Dataset**); (2) estimate the prevalence of Online Fatigue in the Italian academic staff; (3) identify the correlates of Online Fatigue in terms of mental health and psychosomatic symptoms.

## Materials and methods

### Participants and procedure

We conducted a cross-sectional study in the period December 20–31, 2020, using an anonymous web-based survey through the Qualtrics platform, which required about 15 minutes to be completed. A snowball convenience sampling strategy was used to recruit participants (university teachers) through social media–i.e., Facebook, WhatsApp groups of academic staff, and LinkedIn–and institutional emails. The university teachers who had an interest to participate in the study could open a link and receive a detailed cover letter with electronic informed consent. The participants did not receive any form of compensation on participation in this study. Inclusion criteria were: (i) being part of the academic staff, (ii) working in Italy, and (iii) being fluent in Italian.

A total of 322 individuals accessed the online questionnaire, and 307 individuals completed the full survey. The 15 participants who did not fully complete the survey answered less than 50% of the entire questionnaire, thus we decided to exclude them from the study, and only the responses provided by the remaining 307 participants were considered valid. Statistical analyses were carried out with IBM SPSS 25 (release 25.0.0.0).

### Ethical statement

All the procedures conducted were approved by the Ethical Commission of the Department of Psychology of the Università Cattolica del Sacro Cuore of Milan. Informed consent was obtained electronically before data were collected from the participants.

### Development of the Online Fatigue Scale

Some steps were taken to develop the Online Fatigue Scale. First, a scan of the scientific literature was conducted to identify all general scales measuring technology-related stress experience. This analysis led to the identification of two issues, that are the juxtaposition of private life and work-related duties work intensification deriving from the willingness of remote workers to invest greater work effort [23]. In addition, the authors conducted an analysis of the grey literature and press release about the “Zoom fatigue” phenomenon to grasp its phenotypical characteristics from the real world. Crucial dimensions such as the excessive amounts of close-up eye gaze, cognitive load, increased self-evaluation from staring at video of oneself have been considered [33]. Relevant items were pooled by two researchers (i.e., SB and AB). Then, an expert panel (composed of a psychologist, a health psychologist, a psychiatrist, and an expert in human-computer interaction) reviewed the items to evaluate content validity (i.e., the adequacy with which the item pool measured the concept of interest; [34]). Because the expert judges accepted all the items without suggesting modifications, we did not change the original 11-item pool.

### Study measures

The questionnaire included the following measures in the order in which they are presented.

#### Socio-demographic and job-related information

A background information sheet was used to collect participant demographic data (i.e., age, gender, marital status, parental status, residential location), job-related information (i.e., academic position, scientific sector), and information about lifestyle behaviors over the past month.

#### Technology-related experience

A pull of ad-hoc items was used to assess work-related technology usage over the past month. Moreover, Technostress creators were assessed through 11 items derived from the Italian validation of the Technostress Creators Scale [22]: four items for Techno-overload (in this study α =. 837, e.g. of item: “I am forced by technology to work much faster”), three items for Techno-invasion (in this study α =. 806, e.g. of item: “I spend less time with my family due to technology”), and four items for Techno-complexity (in this study α =. 850, e.g. of item: “I do not know enough about technology to handle my job satisfactorily”). In this study, only the Techno-overload and Techno-invasion subscales were used. Participants used a Likert scale from 1 = strongly disagree to 5 = strongly agree. This scale was also used as a concurrent measure for the psychometric testing of the novel “Online Fatigue Scale” specifically developed to assess Online Fatigue.

#### Health status

A checklist of psychosomatic symptoms, adapted from the Copenhagen Psychosocial Questionnaire [35] for this specific study purposes, was developed to assess psychosomatic symptoms that could have been experienced by the academic staff under heavy workloads and distress: participants were asked to report the frequency of these symptoms in the last 4 weeks on a 6-point scale from “never” to “usually.” Moreover, the first item of the Short Form (12) Health Survey (SF-12; [36]) was used to assess participants’ general self-perceived health status (rated on a five-point Likert scale from Excellent to Poor).

#### Psychological wellbeing

The Italian Mental Health Continuum-Short Form (MHC-SF, [37]) consists of 14 items on 6-point scales ranging from 1  =  never to 6 =  everyday. It was used to measure the degree of emotional well-being (EWB, in this study α =. 836, e.g. of item: “During the past month, how often did you feel happy”), social well-being (SWB, in this study α  =  .755, e.g. of item: “During the past month, how often did you feel that you belonged to a community”), and psychological well-being (PWB, in this study, α  =  .834, e.g. of item “During the past month, how often did you feel that you had warm and trusting relationships with others”).

Furthermore, the Perceived Stress Scale (PSS) was used to assess participants’ evaluation of stressful situations over the past month. The PSS is a global measure of stress that is simple to use, and there is an abundance of studies confirming its reliability and validity [38]. Specifically, we used the Italian version of the Short Form PSS (4 items; in this study α  =  .769, e.g. of item “In the last month, how often have you felt that you were unable to control the important things in your life?”). Respondents are asked to rate how often they experienced stressful situations in the previous month on a Likert scale ranging from 0 to 4, where 0 = never and 4 = very often. Two of the PSS-4 items are reverse scored (Q2 and Q3), therefore these variables were recoded. Higher PSS-4 values indicate greater stress.

#### COVID-related perceived distress

To assess the level of emotional balance regarding the COVID-19 emergency we adopted the Patient Health Engagement Scale (PHE-s®) revised form [5]. This measure, developed according to the Patient Health Engagement model, assesses the people health engagement level, defined as the “people’s psychological readiness and sense of mastery to become active players in their own health management and health risk prevention.” Previous studies demonstrated its robust psychometric proprieties, also in other languages. The final score ranges from 0 to 100. Higher values indicate more COVID-related perceived distress.

### Statistical analysis

#### Online Fatigue Scale validation and scoring

Descriptive statistics were computed for each item. Mean, median, and standard deviation, asymmetry and kurtosis have been checked. Values for asymmetry and kurtosis between -2 and +2 were considered acceptable to prove normal distribution [39].

To evaluate the structural and psychometric properties of the Online Fatigue Scale, the sample of 307 participants was randomly divided into two subsamples. No significant differences were found between the two subsamples regarding sex, age and use of the technology. An Exploratory Factor Analysis (EFA) was performed on the first subsample (n = 184, 60% of total sample) to evaluate the factorial structure of the scale, to estimate the factor loadings, to allow for greater accuracy of variable clustering and to minimize the covariance between elements on factors. For the factorial structure of each measure, the Kaiser–Mayer–Olkin measure of sampling adequacy, Bartlett’s test of sphericity (chi-squared and p value), and factor loadings were evaluated. Since the sample was sufficiently large for the number of items, a principal axis method was selected for factor extraction with Promax rotations. Cronbach’s Alpha was calculated to evaluate the internal consistency of each dimension.

Next, to check the adequacy of the items to the identified dimensions, a Confirmatory Factor Analysis (CFA) was run on the second subsample of n = 123 (40% of total sample). The model was estimated using Maximum Likelihood with robust standard errors and evaluated using approximate fit statistics (see Hu and Bentler [40]). These fit statistics included: Root Mean Square Error of Approximation (RMSEA) < 0.08; Confirmatory Fit Index (CFI) > 0.95; and Tucker-Lewis Index (TLI) > 0.90.

Online Fatigue Scale factor scores were evaluated in relation to the first two dimensions of the TCS scale (Techno-Overload and Techno-Invasion) to assess concurrent validity, and in relation to the PSS to assess convergent validity. To obtain a final scoring and to verify the difficulty of each item, a Partial Credit Rasch Model (PCM) was conducted. PCM allows sorting items by increasing difficulty or frequency. A more difficult item (higher value of Location parameter) indicates a situation less frequently experienced by the respondent. The sort of the items allows for an easier determination of the Online Fatigue Scale scoring. Moreover, two mean square statistics (Infit and Outfit Statistics) were computed to check whether the items fitted the expected model. If the data fitted the Rasch Model, the fit statistics should be between 0.5 and 1.5 [41].

#### Socio-demographic characteristics and Online Fatigue Scale

To assess the association between socio-demographic characteristics, technology and videoconferencing platform use, and different Online Fatigue levels, a series of contingency tables were computed. For each categorical variable, the row percentage distribution is presented. For each metric variable, mean and standard deviation in groups were calculated. Pearson’s chi-square was also carried out to reject the null hypothesis that data are randomly distributed across Online Fatigue levels.

#### Health status, well-being, Covid-related perceived stress, and Online Fatigue

A series of univariate ANOVAs, with Online Fatigue dimensions as independent variables, were carried out to explore differences in the frequency of psychosomatic symptoms, behavioral habits, emotional, social and psychological well-being scores, Covid-related perceived distress, and General self-perceived health status among different Online Fatigue levels. P and η^2^ values were calculated.

## Results

### Sociodemographic and professional characteristics

307 academics completed the full survey. Participants’ mean (SD) age was 40.7 (10.1, range 24–70) years, and 72.6% were women. Regarding their academic position, 18.6% were PhD students, 15.6% Research Fellows, 25.0% Assistant Professors, 26.4% Associate or Full Professors, and 14.3% Adjunct Professors. For a more detailed description of the study sample, see Table 1.

**Table 1:** Socio-demographic characteristics and use of technology of the sample (N = 307).

Age (years)	M = 40.7; SD = 10.1
**Gender (% female)**	72.6
**Marital Status (%)**	
Married/Cohabiting	65.8
Widower/Separated/Divorced	4.6
Single/Not Cohabiting	29.6
**Academic Role (%)**	
PhD student	18.6
Research Fellow	15.6
Assistant Professor	25.0
Associate Professor	17.2
Full Professor	9.1
Adjunct Professor	14.3
**Living alone (%)**	
Yes	18.6
No	81.4
**Having Children (%)**	
Yes, even minors	30.9
Yes, only adults	8.5
No	60.6
**Technology Use per day (%)**	
less than 6 hours	16.3
between 6 and 8 hours	35.5
more than 8 hours	48.2
**Online Platforms Use per day (%)**	
less than 2 hours	19.9
between 2 and 4 hours	36.5
more than 4 hours	43.6

### Online Fatigue Scale preliminary validation

Table 2 shows descriptive statistics of the Online Fatigue Scale. Item distributions, as well as the parametric distribution, were normal, since the values of asymmetry and kurtosis were acceptable considering the pre-planned normality range (-2 / +2).

**Table 2:** Descriptive statistics for Online Fatigue Scale items on total sample (N = 307).

	M	SD	Median	Asymmetry	Kurtosis
1) I performed multiple tasks simultaneously (multi-tasking)	4.62	1.29	5.00	-1.08	0.62
2) I struggled to find time off work when I needed it.	4.28	1.39	5.00	-0.81	-0.10
3) I had to give up most of my hobbies.	4.12	1.58	5.00	-0.68	-0.68
4) My life and work needs interfered with each other.	4.27	1.53	5.00	-0.73	-0.53
5) I felt like I had to focus twice more to really understand what was going on	3.76	1.65	4.00	-0.37	-1.11
6) I struggled to allow myself moments of pause in the day between one work activity and another	4.24	1.60	5.00	-0.83	-0.45
7) I felt more socially anxious than usual	3.26	1.77	3.00	0.13	-1.37
8) I found it difficult to get others to listen to me	2.67	1.58	2.00	0.59	-0.80
9) I trusted others less than usual	1.96	1.38	1.00	1.40	0.91
10) I felt like I was having more work meetings than necessary	3.68	1.62	4.00	-0.16	-1.13
11) I worried about my appearance more than I usually do	1.94	1.36	1.00	1.37	0.87

Answering scale from 1 = “Never” to 6 “Every day”.

An EFA with Promax Rotation was performed on the first subsample. The analysis, based on an eigenvalue criterion of > 1.0, yielded two latent factors, with eigenvalues of 4.57 and 1.63 and with 56.32% of explained variance. The correlation matrix had good factorability, Bartlett’s test of sphericity showed that the chi-square was significant at the .0001 level (Chi-square = 1350.03, df = 55, p < 0.001) and the index of Kaiser-Mayer-Olkin measure of sampling adequacy was equal to 0.87. Factor 1 contains items 1, 2, 3, 4, 5, 6, 10 (α = 0.86), Factor 2 contains items 7, 8, 9 and 11 (α = 0.73). The two factors were named *Off-Balance Fatigue* and *Virtual Relations Fatigue*, respectively. Scale scores on each factor were calculated for each respondent by summing ratings for each factor and then dividing by the number of items used to measure it. Table 3 shows the factor loadings and the mean, standard deviations, median, asymmetry, kurtosis and Cronbach’s alphas of both factors, as well the inter-scale correlations. Off-Balance and Virtual Relations Fatigue dimensions share a moderate, positive, and significant correlation (r = 0.50, *p*<0.001).

**Table 3:** Subsample 1 (n = 184): Factor loadings, descriptive statistics, Cronbach’s Alphas, and inter-scale correlation of Online Fatigue Scale (Off-Balance and virtual relations fatigue).

	Factor	
	1	2
I performed multiple tasks simultaneously (multi-tasking)	0.39	
I struggled to find time off work when I needed it.	0.89	
I had to give up most of my hobbies.	0.88	
My life and work needs interfered with each other.	0.74	
I felt like I had to focus twice more to really understand what was going on	0.40	
I struggled to allow myself moments of pause in the day between one work activity and another	0.80	
I felt more socially anxious than usual		0.70
I found it difficult to get others to listen to me		0.79
I trusted others less than usual		0.68
I felt like I was having more work meetings than necessary	0.48	
I worried about my appearance more than I usually do		0.39
Mean	4.14	2.46
Standard Deviation	1.12	1.13
Median	4.43	2.25
Asymmetry	-0.61	0.74
Kurtosis	-0.35	-0.07
Cronbach’s Alpha	0.86	0.73
Inter-Factors correlation	0.50	-

A CFA was then run on the second subsample. Results showed that the proposed solution provided an acceptable fit to the data, CFI = 0.951, TLI = 0.934, RMSEA = 0.072 (LO90 = 0.055, HI90 = 0.089) and factor loadings ranging from 0.38 to 0.85, with median standardized loading = 0.71.

Table 4 shows the correlations between the two dimensions of the Online Fatigue Scale and the related measures for concurrent (TCS scale) and convergent (PSS scale) validity.

**Table 4:** Correlation between Online Fatigue scale and TCS and PSS.

	TCS				
	Techno-Overload	Techno-Invasion	PSS	
Off-Balance Fatigue	.558*	.555*	.407*	
Virtual Relations Fatigue	.404*	.397*	.529*	

* p<0.001.

Table 5 shows the results of the Rasch Analysis. The location of each item represents the estimate for the item difficulty expressed in logits; SEM is the standard error of measurement in estimation of the item difficulty; Infit and Outfit are measures of item fit. Items are sorted by difficulty parameter: a less difficult item (i.e., lower location score) indicates that the situation described in the item is more frequent and more experienced by the respondents. On the other hand, a more difficult item (i.e., higher location score) indicates that the situation described in the item is less frequent and less experienced by the respondents. The item statistics ranged from .666 to 1.269 for the infit statistic and from .653 to 1.354 for the outfit statistic. These values indicate an acceptable fit of the Rasch Model.

**Table 5:** Partial credit model and item fit statistics.

Item	Location	SE	Oufit Statistic	Infit Statistic
I performed multiple tasks simultaneously (multi-tasking)	-0.597	0.266	1.341	1.269
I struggled to find time off work when I needed it	-0.298	0.232	0.757	0.707
My life and work needs interfered with each other	-0.281	0.223	0.653	0.666
I struggled to allow myself moments of pause in the day between one work activity and another	-0.165	0.233	0.685	0.699
I had to give up most of my hobbies	-0.131	0.221	0.788	0.758
I felt like I was having more work meetings than necessary	0.056	0.201	1.097	1.087
I felt like I had to focus twice more to really understand what was going on	0.092	0.209	0.846	0.826
I felt more socially anxious than usual	0.300	0.208	0.989	0.979
I found it difficult to get others to listen to me	0.666	0.222	1.024	0.938
I trusted others less than usual	1.128	0.287	1.342	0.910
I worried about my appearance more than I usually do	1.140	0.294	1.354	1.244

The different choice of scoring between Off-Balance and Virtual Relations Fatigue subscales was due to the fact that the items included in the Virtual Relations Fatigue subscale were more difficult than the Off-Balance Fatigue items. Specifically, the least difficult items (i.e., those related to the most frequently experienced situations) according to the results of the Rasch Model were those included in the Off-Balance Fatigue subscale (i.e., items 1, 2, 3, 4, 5, 6, 10). On the other hand, the most difficult items (i.e., those related to the least frequently experienced situations) were those included in the Virtual Relations Fatigue subscale (i.e., items 7, 8, 9, 11). Hence the reason for making different scorings for each subscale. This is also understandable if one considers that the situations presented in the Off-Balance Fatigue dimension (which refer to a general sense of lack of time/mental space for family and self-care) are more likely to be experienced in everyday life compared to the situations presented in the Virtual Relations Fatigue subscale (which refer to stressful experiences during virtual interactions with others).

In view of the above, the Off-Balance Fatigue subscale was recoded into 3 levels: low (0 or 1 situation of Off-Balance Fatigue every day/almost every day), medium (from 2 to 4 situations of Off-Balance Fatigue every day/almost every day), and high (from 5 to 7 different situations of Off-Balance Fatigue every day/almost every day). The Virtual Relations Fatigue subscale was recoded into 2 levels: low (no situations of fatigue experienced every day/almost every day) and high (at least one situation of fatigue experienced every day/almost every day).

As regards Off-Balance Fatigue, 27.4% of the participants reported a low level, 38.4% a medium level, and 34.2% a high level. Regarding Virtual Relations Fatigue, 61.9% of the participants showed a low level and 38.1% a high level.

### Socio-demographic characteristics and Online Fatigue

The results of the contingency table to assess the association between socio-demographic characteristics, technology and use of communication platforms, and different Off-Balance and Virtual Relations Fatigue levels are reported in Tables 6 and 7, respectively. For each variable, the row percentage distribution and Chi Square test have been reported.

**Table 6:** Results of contingency table–Off balance fatigue subscale.

Socio-demographic variables	Answers	Off-Balance Fatigue level			Total
		*low*	*medium*	*high*	
Age	M (SD)	40.8 (10.6)	40.0 (10.1)	41.3 (9.7)	40.7 (10.1)
Gender	male	34.2%	41.8%	24.1%	100%
*χ*^*2*^ *= 5*,*862(df = 2)*, *p <* .*1*	female	24.7%	36.8%	38.6%	100%
Academic Role	PhD / Research Fellow / Adjunct Prof.	28.2%	39.6%	32.2%	100%
*χ*^*2*^ *= 2*.*261(df = 4)*, *p =* .*688*	Assistant Professor	29.9%	39.0%	31.2%	100%
	Associate / Full Professor	23.5%	35.8%	40.7%	100%
Marital status	married / cohabitating	26.2%	38.1%	35.6%	100%
*χ*^*2*^ *= 1*.*698(df = 4)*, *p =* .*791*	widowed / separated / divorced	21.4%	35.7%	42.9%	100%
	single / not cohabitating	30.8%	39.6%	29.7%	100%
Living alone	yes	35.1%	40.4%	24.6%	100%
*χ*^*2*^ *= 3*.*496(df = 2)*, *p =* .*174*	no	25.6%	38.0%	36.4%	100%
Having children	yes, including minors	20.0%	35.8%	44.2%	100%
*χ*^*2*^ *= 8*.*012(df = 4)*, *p <* .*1*	yes, only adults	26.9%	34.6%	38.5%	100%
	no	31.2%	40.3%	28.5%	100%
Tecnhology Use per day	less than 6 hours	42.0%	46.0%	12.0%	100%
*χ*^*2*^ *= 29*.*539(df = 4)*, *p <* .*001*	between 6 and 8 hours	36.7%	33.9%	29.4%	100%
	more than 8 hours	15.5%	39.2%	45.3%	100%
Videoconferencing Platform Use per day *χ*^2^ *= 23*.*277(df = 4)*, *p <* .*001*	less than 2 hours	47.5%	34.4%	18.0%	100%
	between 2 and 4 hours	26.8%	42.9%	30.4%	100%
	more than 4 hours	18.7%	36.6%	44.8%	100%

**Table 7:** Results of contingency table–Virtual relations fatigue subscale.

Socio-demographic variables	Answers	Virtual Relations Fatigue level		Total
		*low*	*high*	
Age	M (SD)	41.2 (10.3)	39.8 (9.7)	40.7 (10.1)
Gender	male	64.6%	35.4%	100%
*χ*^*2*^ *=* .*315(df = 1)*, *p =* .*574*	female	61.0%	39.0%	100%
Academic Role	PhD / Research Fellow / Adjunct Prof.	59.7%	40.3%	100%
*χ*^*2*^ *=* .*637(df = 2)*, *p =* .*727*	Assistant Professor	64.9%	35.1%	100%
	Associate / Full Professor	63.0%	37.0%	100%
Marital status	married / cohabitating	65.3%	34.7%	100%
*χ*^*2*^ *= 3*.*018(df = 2)*, *p =* .*221*	widowed / separated / divorced	57.1%	42.9%	100%
	single / not cohabiting	54.9%	45.1%	100%
Living alone	yes	56.1%	43.9%	100%
*χ*^*2*^ *=* .*981(df = 1)*, *p =* .*322*	no	63.2%	36.8%	100%
Having children	yes, including minors	64.2%	35.8%	100%
*χ*^*2*^ *=* .*320(df = 2)*, *p =* .*852*	yes, only adults	61.5%	38.5%	100%
	no	60.8%	39.2%	100%
Technology Use per day	less than 6 hours	62.0%	38.0%	100%
*χ*^*2*^ *=* .*444(df = 2)*, *p =* .*801*	between 6 and 8 hours	64.2%	35.8%	100%
	more than 8 hours	60.1%	39.9%	100%
Videoconferencing Platform Use per day *χ*^2^ *= 1*.*009(df = 2)*, *p =* .*604*	less than 2 hours	67.2%	32.8%	100%
	between 2 and 4 hours	61.6%	38.4%	100%
	more than 4 hours	59.7%	40.3%	100%

Results showed a moderate significant association between a high level of Off-Balance Fatigue and the use of technology and videoconferencing platforms. A high level of Off-Balance Fatigue was reported by 45.3% of the participants who used technology more than 8 hours per day and by 44.8% of those who used videoconferencing platforms more than 4 hours per day. High levels of Off-Balance Fatigue were also more frequently experienced by women and people who had minor children, even if differences were not significant. Off-Balance Fatigue levels did not vary across different age and academic role.

A high level of Virtual Relations Fatigue was reported by 43.9% of participants who lived alone and by 45.1% of those who were single / not cohabiting. However, these associations were not significant. In addition, Virtual Relations Fatigue levels did not vary across different age, academic role and gender profiles, as well as across different use of technology and videoconferencing platforms.

### Physical and psychological correlates of Online Fatigue

Tables 8–10 show descriptive statistics (Table 8) of the physical and psychological correlates and the results of the univariate ANOVA performed to compare the frequency of psychosomatic symptoms (items adapted from the Copenhagen Psychosocial Questionnaire), general self-perceived health status (first item of the SF-12), behavioral health habits, Emotional, Social and Psychological well-being score, and Covid-related perceived distress in participants with low vs. medium vs. high Off-Balance Fatigue (see Table 9), and low vs. high Virtual Relations Fatigue (see Table 10).

**Table 8:** Descriptive statistics for physical and psychological correlates on total sample (N = 307).

	M	SD	Median	Asymmetry	Kurtosis
Chest pains*	2.07	1.46	1	1.11	0.05
Palpitations*	2.51	1.61	2	0.63	-0.84
Dizziness*	1.83	1.33	1	1.48	1.23
Muscle tension*	4.04	1.87	4	-0.32	-0.91
Sleep disturbances*	3.92	1.82	4	-0.13	-1.03
Gastro-intestinal disorders*	2.90	1.85	2	0.56	-0.89
Irritability and mood alteration*	4.20	1.60	4	-0.18	-0.55
Altered eating habits*	4.34	1.52	4	-0.54	0.08
Visual disturbances*	3.71	1.87	4	-0.05	-1.16
Dysphonia and vocal tract discomfort*	2.79	1.77	2	0.57	-0.86
General Health (SF-12)**	2.50	0.87	3	0.25	0.16
I drank more than 3 cups of caffeinated beverages per day*	3.12	2.07	2	0.27	-1.64
I did physical activity (at home or outdoors)*	2.70	1.49	2	0.42	-0.91
I have practiced yoga / pilates or other similar disciplines*	1.71	1.27	1	1.67	1.66
I practiced meditation*	1.33	0.96	1	3.29	10.31
Emotional Well-Being***	4.04	1.06	4.33	-0.69	0.21
Social Well-Being***	2.67	0.97	2.60	0.51	0.06
Psychological Well-Being***	4.09	0.96	4.17	-0.53	0.07
Covid-related perceived distress****	52.24	17.80	51.93	0.02	0.06

* Answering scale from 1 = “Never” to 6 “Usually”.

** Answering scale from 1 =“Excellent” to 5 “Poor”.

*** Scores from 1 to 6.

**** Scores from 0 to 100.

**Table 9:** Results of Anova–Off balance fatigue levels and correlates.

	Off Balance level			F_2,304_	p	η^2^
	low	medium	high			
Chest pains*	1.57	1.99	2.54	11.239	0.000	0.069
Palpitations*	1.96	2.46	3.00	10.297	0.000	0.063
Dizziness*	1.48	1.68	2.28	10.304	0.000	0.063
Muscle tension*	3.43	3.74	4.88	18.408	0.000	0.108
Sleep disturbances*	3.23	3.75	4.65	16.462	0.000	0.098
Gastro-intestinal disorders*	2.31	2.84	3.44	9.216	0.000	0.057
Irritability and mood alteration*	3.36	4.02	5.07	33.959	0.000	0.183
Altered eating habits*	2.80	3.19	4.00	12.639	0.000	0.077
Visual disturbances*	2.93	3.64	4.41	16.321	0.000	0.097
Dysphonia and vocal tract discomfort*	2.43	2.55	3.33	8.144	0.000	0.051
General Health (SF-12)**	2.46	2.42	2.61	1.455	0.235	0.009
I drank more than 3 cups of caffeinated beverages per day*	2.86	2.94	3.53	3.267	0.039	0.021
I did physical activity (at home or outdoors)*	3.11	2.78	2.30	7.521	0.001	0.047
I have practiced yoga / pilates or other similar disciplines*	1.80	1.78	1.55	1.191	0.305	0.008
I practiced meditation*	1.25	1.39	1.31	0.528	0.591	0.003
Emotional Well-Being***	4.25	4.09	3.82	4.015	0.019	0.026
Social Well-Being***	2.77	2.74	2.50	2.291	0.103	0.015
Psychological Well-Being***	4.29	4.14	3.87	4.583	0.011	0.029
Covid-related perceived distress****	56.39	52.88	48.19	5.211	0.006	0.033

* Answering scale from 1 = “Never” to 6 “Usually”.

** Answering scale from 1 =“Excellent” to 5 “Poor”.

*** Scores from 1 to 6.

**** Scores from 0 to 100.

**Table 10:** Results of Anova–Virtual relations fatigue levels and correlates.

	Virtual Relations level		F_1,305_	p	η^2^
	low	high			
Chest pains*	1.81	2.48	15.839	0.000	0.049
Palpitations*	2.11	3.16	34.479	0.000	0.102
Dizziness*	1.57	2.24	19.347	0.000	0.060
Muscle tension*	3.87	4.32	4.095	0.044	0.013
Sleep disturbances*	3.58	4.46	17.932	0.000	0.056
Gastro-intestinal disorders*	2.56	3.44	17.228	0.000	0.053
Irritability and mood alteration*	3.59	5.18	93.492	0.000	0.235
Altered eating habits*	3.01	3.92	20.612	0.000	0.063
Visual disturbances*	3.49	4.06	6.894	0.009	0.022
Dysphonia and vocal tract discomfort*	2.54	3.19	10.102	0.002	0.032
General Health (SF-12)**	2.47	2.53	0.300	0.584	0.001
I drank more than 3 cups of caffeinated beverages per day*	2.83	3.59	10.020	0.002	0.032
I did physical activity (at home or outdoors)*	2.87	2.43	6.650	0.010	0.021
I have practiced yoga / pilates or other similar disciplines*	1.76	1.62	0.984	0.322	0.003
I practiced meditation*	1.31	1.35	0.124	0.725	0.000
Emotional Well-Being***	4.31	3.60	36.371	0.000	0.107
Social Well-Being***	2.83	2.41	14.022	0.000	0.044
Psychological Well-Being***	4.31	3.73	29.130	0.000	0.087
Covid-related perceived distress****	55.96	46.18	23.478	0.000	0.071

* Answering scale from 1 = “Never” to 6 “Usually”.

** Answering scale from 1 =“Excellent” to 5 “Poor”.

*** Scores from 1 to 6.

**** Scores from 0 to 100.

**Fig 1:**
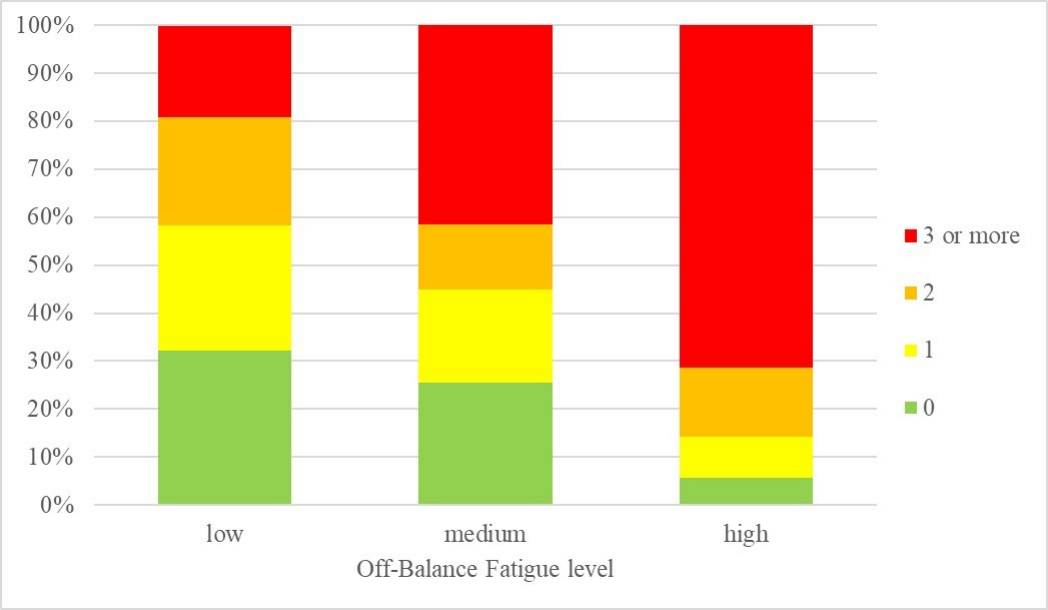
Number of psychosomatic symptoms per Off-Balance fatigue level.

**Fig 2:**
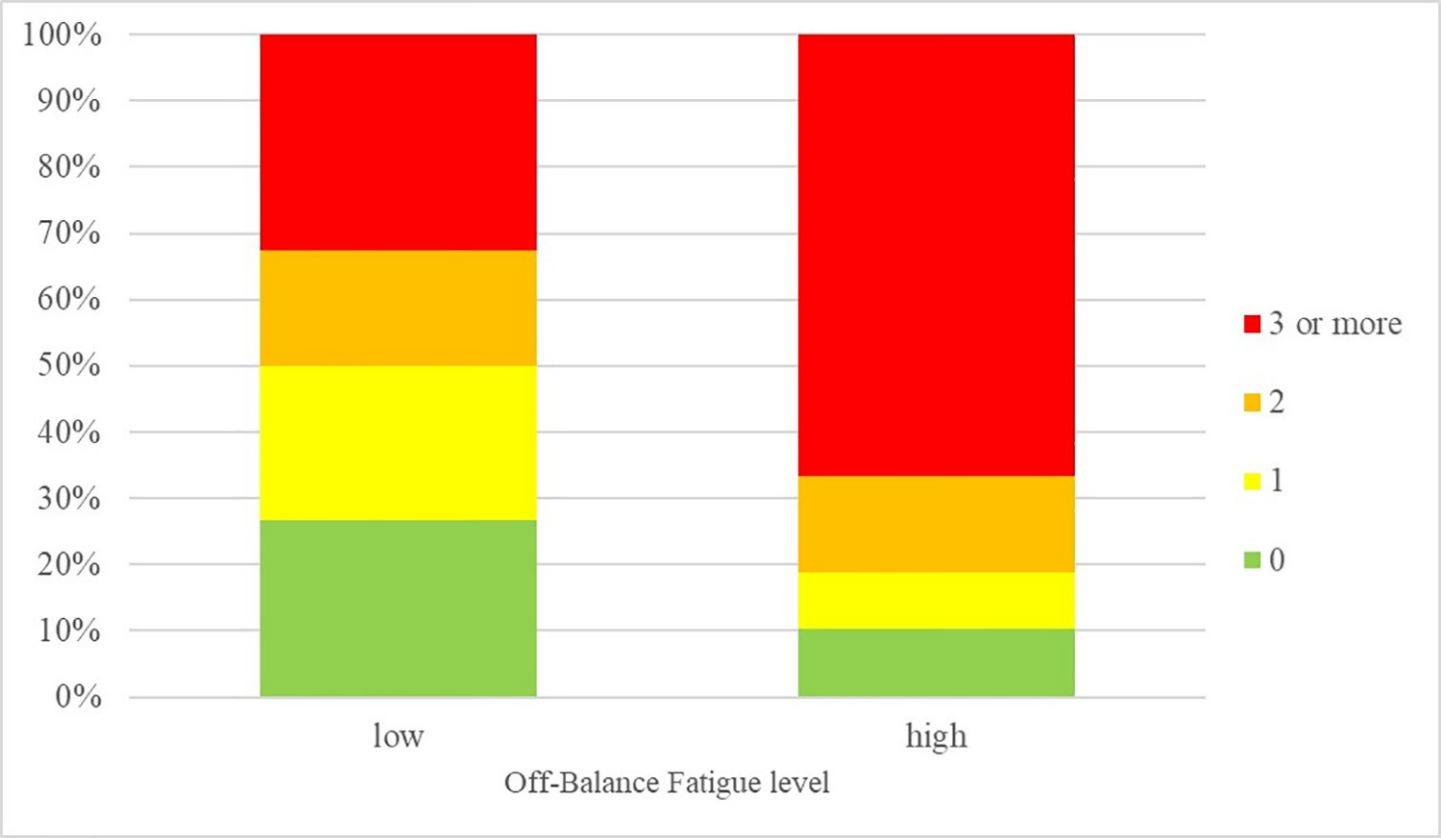
Number of psychosomatic symptoms per Virtual Relations fatigue level.

ANOVA results showed a significant main effect of Online Fatigue on all psychosomatic symptoms. In general, a high level of Off-Balance and Virtual Relations Fatigue were associated with a higher frequency of psychosomatic symptoms (see Figs 1 and 2): especially muscle tension, irritability, and visual disturbances in participants with high Off-Balance Fatigue vs. participants with medium-low Off-Balance Fatigue, and palpitations, irritability and mood alterations in participants with high vs. low Virtual Relations Fatigue (see the η^2^ values reported in Tables 9 and 10). Off-Balance Fatigue had also a significant effect on some unhealthy habits, on Emotional and Psychological Well-Being, and on Covid-related perceived distress. In addition, Virtual Relations Fatigue had a significant effect on some behavioral health habits and a significant effect on every subscale of the Well-Being Scale and on Covid-related perceived distress.

## Discussion

In this cross-sectional study, we examined a new form of pandemic-related fatigue, referred to as Online Fatigue. We decided to examine Online Fatigue among Italian academics because they experienced a remarkable shift to homeworking, which involved a massive use of technologies for multiple activities (including work meetings and online teaching). Considering that the association between use of technology and stress has been demonstrated in several studies [24, 26], and that fatigue has been acknowledged as an important component of people’s subjective experience during the current pandemic [1, 42], we wanted to estimate the prevalence of Online Fatigue in this population, which indeed has its own specificities. At the same time, we considered our sample as a prototypical population, whose work life may reflect those of many other homeworkers during the pandemic.

The conceptual definition of Online Fatigue was provided after careful review of the extant literature on technostress (including technostress measures), as well as of the published material (press release and very few articles) on the so called “Zoom fatigue” phenomenon. Based on this definition, we developed an 11-item assessment tool–the Online Fatigue Scale–that showed good psychometric properties. The first step of this study aimed to evaluate the structural and psychometric properties of the Online Fatigue Scale, and its association with concurrent and convergent measures.

EFA and CFA conducted on two independent subsamples showed a two-factor structure, named Off-Balance Fatigue (related to a stressful experience linked to impaired work-life balance, due for instance to difficulties finding the time for hobbies or simply for a break during the day, as well as to excessive workload) and Virtual Relations Fatigue (related to a stressful experience linked to interacting with other using technology), with a good internal reliability as indicated by satisfactory Cronbach’s Alpha. The Rasch Analysis confirmed the importance (in terms of fitting) of each item of the scale by using the Infit and Outfit Statistics. Moreover, the Rasch Analysis allowed to rank the items based on their difficulty, which was useful for the scoring procedure.

Finally, to assess the concurrent validity, the Online Fatigue Scale factor scores were evaluated in relation to two dimensions of the TCS scale (Techno-Overload and Techno-Invasion), because these dimensions refer to the feeling of being forced by ICTs to change work habits (work faster and longer), and to the subjective perception that ICTs blur the boundaries between work and private life. To assess convergent validity, the Online Fatigue Scale factor scores were evaluated in relation to the PSS Scale, as previous studies already demonstrated the positive association between these dimensions and behavioral strain, according to the transaction-based model of stress [43], showing moderate significant correlations. These analyses showed that the 11-item version of the Online Fatigue Scale can be considered a reliable and valid instrument to assess Off Balance and Virtual Relations Fatigue among Italian academics.

On the one hand, technology enabled the academic staff to work from home and continue doing their jobs despite stay-at-home orders (which also contributed to guarantee physical distancing). On the other hand, our results showed that, in our sample, Off-Balance Fatigue was associated with very frequent use of technology and communication platforms, especially in relation to being a woman and having minor children. These findings are understandable if one considers that, due to the COVID containment measures, work and private life bled into one another, with impaired balance between professional and home duties. In this regard, women and people with very young children may be particularly at risk of experiencing an “invasion” of technologies in their private life during homeworking.

Academic work is not standardizable in terms of work hours, but all types of previous work-life balance arrangements might have been completely disrupted by the current pandemic. In these difficult times, universities should promote the separation between work and private life, for instance by planning periodic downtime to step away from technology, and invest in the psychological and physical health of the academic staff by encouraging self-care practices, including physical activities [44].

During in-person meetings, people exchange either verbal or non-verbal information, and thus communicate implicit attitudes and feelings towards their colleagues, which are also related to the quality of interactions. Videoconferencing entails a greater effort to cognitively process non-verbal cues (such as postures and body movements, haptic communication, and proxemics) [37] and leads to a paradoxical disconnection between participants’ minds and bodies. This sense of placelessness directly affects our episodic memory and our personal and professional identity [42], with negative psychological consequences in terms of sense of exhaustion, social anxiety and stress. Moreover, joint attention–i.e., the ability to have a shared focus on a common object, which entails coordinating with other [45]–is limited in videoconferencing due to the impossibility of using eye contact and glance exchange, with decreased group engagement, along with a greater effort to cooperate successfully with others [46].

In our study, both Off-Balance and Virtual Relations Fatigue were associated with a higher frequency of psychosomatic symptoms. In particular, irritability was a common symptom, whereas Off-Balance Fatigue was more associated with physical tension, as confirmed by the literature on the effects of technostress [26]. On the other hand, Virtual Relations Fatigue was associated with mood alterations, which highlighted the role of the affective dimension involved in interpersonal online communication [47].

Moreover, although the two Online Fatigue subscales were not associated with the participants’ general health status, they were significantly associated with Covid-related distress. These findings indicated that Online Fatigue is specifically related to the pandemic, an unprecedented global challenge that has been revolutionizing the private and work lives of many people.

## Conclusions and limitations

Our study contributed to detect the presence of Online Fatigue among Italian academics as a consequence of the new work conditions imposed by the pandemic. This complex situation needs to be addressed at different levels. On the one hand, greater attention should be paid to the balance between working and personal life, by defining spaces (even inside academic’s home) and working times, and thus sustaining an ethics of care [48]. University administrators could play an important role in addressing the physical and mental health of their academic staff, and to supply indications for health policy managers to implement effective interventions to relieve the mental burden and reduce the fatigue level, as well as to contribute to the construction of safe work environments. On the other hand, our results highlighted the importance of supporting self-care strategies, which include the possibility of exploiting the potential of interventions based on meditation, relaxation and positive psychology practices, in a personalized way [49–51]. Finally, it is important to recognize the chances offered by advanced videoconferencing tools, that allow to place participants’ live video images into a fixed position within a digital setting to increase the feeling of sharing a common space, or to use virtual reality social platforms to better support authentic relationships [42, 52]. This study has also some limitations: we measured a specific population’s views at a specific point in time; their beliefs and attitudes reflect the information available at the time and therefore are not stable. Second, results were self-reported. Measurement errors and social desirability bias may exist although the study was anonymous. Finally, the sample was not statistically representative. Moreover, the sample size was low and participants were unbalanced by gender. Further studies should be conducted to corroborate the validity of this results.

## Supporting information

S1 Dataset (SAV)

## References

[1] Wiederhold BK. Connecting through Technology during the Coronavirus Disease 2019 Pandemic: Avoiding “zoom Fatigue.” Vol. 23, Cyberpsychology, Behavior, and Social Networking. 2020. 10.1089/cyber.2020.29188.bkw 32551981

[2] Centers for Disease Control and Prevention. No Title [Internet]. Coronavirus (COVID 19). 2020. Available from: https://www.cdc.gov/coronavirus/2019-ncov/index.html

[3] Asmundson GJG, Taylor S. How health anxiety influences responses to viral outbreaks like COVID-19: What all decision-makers, health authorities, and health care professionals need to know. Vol. 71, Journal of Anxiety Disorders. 2020. 10.1016/j.janxdis.2020.102211 PMC7271220 32179380

[4] Courtet P, Olie E, Debien C, Vaiva G. Keep socially (but not physically) connected and carry on: Preventing suicide in the age of COVID-19. Vol. 81, Journal of Clinical Psychiatry. 2020. doi: 10.4088/JCP.20com13370 32297718

[5] Graffigna G, Barello S, Savarese M, Palamenghi L, Castellini G, Bonanomi A, et al. Measuring Italian citizens’ engagement in the first wave of the COVID-19 pandemic containment measures: A cross-sectional study. PLoS One. 2020;15(9 September). doi: 10.1371/journal.pone.0238613 32915822 PMC7485890

[6] Tull MT, Edmonds KA, Scamaldo KM, Richmond JR, Rose JP, Gratz KL. Psychological Outcomes Associated with Stay-at-Home Orders and the Perceived Impact of COVID-19 on Daily Life. Psychiatry Res. 2020;289. doi: 10.1016/j.psychres.2020.113098 32434092 PMC7252159

[7] WHO. No Title [Internet]. 2020. Available from: https://apps.who.int/iris/bitstream/handle/10665/337574/WHO-EURO-2020-1573-41324-56242-eng.pdf?sequence=1&isAllowed=y

[8] Barello S, Palamenghi L, Graffigna G. Burnout and somatic symptoms among frontline healthcare professionals at the peak of the Italian COVID-19 pandemic. Psychiatry Res. 2020;290. 10.1016/j.psychres.2020.113129 PMC7255285 32485487

[9] Converso D, Bruno A, Capone V, Colombo L, Falco A, Galanti T, et al. Working during a pandemic between the risk of being infected and/or the risks related to social distancing: First validation of the saph@w questionnaire. Int J Environ Res Public Health. 2021;18(11). doi: 10.3390/ijerph18115986 34199612 PMC8199693

[10] Warren MA, Bordoloi SD. When COVID-19 exacerbates inequities: The path forward for generating wellbeing. Int J Wellbeing. 2020;10(3).

[11] Harju LK, Rokka J, Lopes MM, Airoldi M, Raïes K. Employee Well-Being Profiles During COVID-19 Lockdown: A Latent Profile Analysis of French and UK Employees. Front Psychol. 2021;12. doi: 10.3389/fpsyg.2021.645300 34177699 PMC8219910

[12] Prasad KD V, Vaidya RW, Mangipudi MR. Effect of Occupational Stress and Remote Working on Psychological Wellbeing of Employees: An Empirical Study during Covid-19 Pandemic with Reference to Information Technology Industry around Hyderabad. Indian J Commer Manag Stud. 2020;2.

[13] Saxena A, Gautam SS. Employee mental well-being amidst Covid-19: Major stressors and distress. J Public Aff. 2020; doi: 10.1002/pa.2552 33349740 PMC7744868

[14] Petitta L, Probst TM, Ghezzi V, Barbaranelli C. Emotional contagion as a trigger for moral disengagement: Their effects on workplace injuries. Saf Sci. 2021;140.

[15] Cullen W, Gulati G, Kelly BD. Mental health in the COVID-19 pandemic. QJM. 2020;113(5). 10.1093/qjmed/hcaa110 PMC7184387 32227218

[16] Pagnini F, Bonanomi A, Tagliabue S, Balconi M, Bertolotti M, Confalonieri E, et al. Knowledge, Concerns, and Behaviors of Individuals During the First Week of the Coronavirus Disease 2019 Pandemic in Italy. JAMA Netw open. 2020;3(7). 10.1001/jamanetworkopen.2020.15821 PMC7382000 32706385

[17] Nicola M, Alsafi Z, Sohrabi C, Kerwan A, Al-Jabir A, Iosifidis C, et al. The socio-economic implications of the coronavirus pandemic (COVID-19): A review. Vol. 78, International Journal of Surgery. 2020. doi: 10.1016/j.ijsu.2020.04.018 32305533 PMC7162753

[18] Wang C, Pan R, Wan X, Tan Y, Xu L, Ho CS, et al. Immediate psychological responses and associated factors during the initial stage of the 2019 coronavirus disease (COVID-19) epidemic among the general population in China. Int J Environ Res Public Health. 2020;17(5). doi: 10.3390/ijerph17051729 32155789 PMC7084952

[19] Ream E, Richardson A. Fatigue: A concept analysis. Int J Nurs Stud. 1996;33(5). doi: 10.1016/0020-7489(96)00004-1 8886902

[20] Lee J. A Neuropsychological Exploration of Zoom Fatigue. Psychiatr Times. 2020;37(11).

[21] Morris B. Why Does Zoom Exhaust You? Science Has an Answer. Wall Str J—Online Ed. 2020;

[22] Molino M, Ingusci E, Signore F, Manuti A, Giancaspro ML, Russo V, et al. Wellbeing costs of technology use during Covid-19 remote working: An investigation using the Italian translation of the technostress creators scale. Sustain. 2020;12(15).

[23] Palumbo R. Let me go to the office! An investigation into the side effects of working from home on work-life balance. Int J Public Sect Manag. 2020;33(6–7).

[24] Tarafdar M, Tu Q, Ragu-Nathan T. Impact of technostress on end-user satisfaction and performance. J Manag Inf Syst. 2010;27(3).

[25] Tarafdar M, Tu Q, Ragu-Nathan BS, Ragu-Nathan TS. The impact of technostress on role stress and productivity. J Manag Inf Syst. 2007;24(1).

[26] La Torre G, Esposito A, Sciarra I, Chiappetta M. Definition, symptoms and risk of techno-stress: a systematic review. Vol. 92, International Archives of Occupational and Environmental Health. 2019. doi: 10.1007/s00420-018-1352-1 30196317

[27] Arnetz BB, Wiholm C. Technological stress: Psychophysiological symptoms in modern offices. In: Journal of Psychosomatic Research. 1997. doi: 10.1016/s0022-3999(97)00083-4 9263929

[28] Ragu-Nathan TS, Tarafdar M, Ragu-Nathan BS, Tu Q. The consequences of technostress for end users in organizations: Conceptual development and validation. Inf Syst Res. 2008;19(4).

[29] Ayyagari R, Grover V, Purvis R. Technostress: Technological antecedents and implications. MIS Q Manag Inf Syst. 2011;35(4).

[30] Husky MM, Kovess-Masfety V, Swendsen JD. Stress and anxiety among university students in France during Covid-19 mandatory confinement. Compr Psychiatry. 2020;102. doi: 10.1016/j.comppsych.2020.152191 32688023 PMC7354849

[31] Nania T, Dellafiore F, Caruso R, Barello S. Risk and protective factors for psychological distress among Italian university students during the COVID-19 pandemic: The beneficial role of health engagement. International Journal of Social Psychiatry. 2020. doi: 10.1177/0020764020945729 32721256

[32] Ye Z, Yang X, Zeng C, Wang Y, Shen Z, Li X, et al. Resilience, Social Support, and Coping as Mediators between COVID-19-related Stressful Experiences and Acute Stress Disorder among College Students in China. Appl Psychol Heal Well-Being. 2020;12(4). 10.1111/aphw.12211 PMC7405224 32666713

[33] Bailenson JN. Nonverbal overload: A theoretical argument for the causes of Zoom fatigue. Technol Mind, Behav. 2021;2(1).

[34] Boateng GO, Neilands TB, Frongillo EA, Melgar-Quiñonez HR, Young SL. Best Practices for Developing and Validating Scales for Health, Social, and Behavioral Research: A Primer. Front Public Heal. 2018;6. 10.3389/fpubh.2018.00149 PMC6004510 29942800

[35] Kristensen TS, Hannerz H, Høgh A, Borg V. The Copenhagen Psychosocial Questionnaire—A tool for the assessment and improvement of the psychosocial work environment. Scand J Work Environ Heal. 2005;31(6). doi: 10.5271/sjweh.948 16425585

[36] Burdine JN, Felix MR, Abel AL, Wiltraut CJ, Musselman YJ. The SF-12 as a population health measure: an exploratory examination of potential for application. Health Serv Res. 2000;35(4). 11055454 PMC1089158

[37] Petrillo G., Capone V., Caso D., & Keyes CL. The Mental Health Continuum–Short Form (MHC–SF) as a measure of well-being in the Italian context. Soc Indic Res. 2015;121(1):291–312.

[38] Mondo M, Sechi C, Cabras C. Psychometric evaluation of three versions of the Italian Perceived Stress Scale. Curr Psychol. 2019;

[39] George D, Mallery P. SPSS for Windows Step by Step: A Simple Guide and Reference Fourth Edition. Bost Pearson Educ Inc. 2010;

[40] Hu LT, Bentler PM. Cutoff criteria for fit indexes in covariance structure analysis: Conventional criteria versus new alternatives. Struct Equ Model. 1999;6(1).

[41] Wright BD, Linacre JM. Reasonable mean-square fit values. Rasch Meas Trans. 1994;8(3).

[42] Riva G., Wiederhold B. K., & Mantovani F. Surviving COVID-19: The Neuroscience of Smart Working and Distance Learning. Cyberpsychology, Behav Soc Netw. 2021;24(2):79–85. doi: 10.1089/cyber.2021.0009 33577414

[43] Lazarus RS. Psychological stress in the workplace. Vol. 11, Journal of UOEH. 1989. 2664957

[44] Maugeri G, Castrogiovanni P, Battaglia G, Pippi R, D’Agata V, Palma A, et al. The impact of physical activity on psychological health during Covid-19 pandemic in Italy. Heliyon. 2020;6(6). doi: 10.1016/j.heliyon.2020.e04315 32613133 PMC7311901

[45] Mundy P, Newell L. Attention, joint attention, and social cognition. Curr Dir Psychol Sci. 2007;16(5). 10.1111/j.1467-8721.2007.00518.x PMC2663908 19343102

[46] Kraut RE, Fussell S, Brennan S, Siegel J. Understanding effects of proximity on collaboration: Implications for technologies to support remote collaborative work. Distrib Work. 2002;

[47] Cipresso P, Villani D, Repetto C, Bosone L, Balgera A, Mauri M, et al. Computational Psychometrics in Communication and Implications in Decision Making. Comput Math Methods Med. 2015;2015. doi: 10.1155/2015/985032 26339285 PMC4538966

[48] Corbera E, Anguelovski I, Honey-Rosés J, Ruiz-Mallén I. Academia in the Time of COVID-19: Towards an Ethics of Care. Plan Theory Pract. 2020;21(2).

[49] Linardon J, Cuijpers P, Carlbring P, Messer M, Fuller-Tyszkiewicz M. The efficacy of app-supported smartphone interventions for mental health problems: a meta-analysis of randomized controlled trials. World Psychiatry. 2019;18(3). doi: 10.1002/wps.20673 31496095 PMC6732686

[50] Carissoli C, Villani D, Riva G. Does a meditation protocol supported by a mobile application help people reduce stress? suggestions from a controlled pragmatic trial. Cyberpsychology, Behav Soc Netw. 2015;18(1). doi: 10.1089/cyber.2014.0062 25584730

[51] Gaggioli A, Villani D, Serino S, Banos R, Botella C. Editorial: Positive technology: Designing e-experiences for positive change. Vol. 10, Frontiers in Psychology. 2019. doi: 10.3389/fpsyg.2019.01571 31338053 PMC6629820

[52] Wiederhold BK. Embodiment Empowers Empathy in Virtual Reality. Vol. 23, Cyberpsychology, Behavior, and Social Networking. 2020. doi: 10.1089/cyber.2020.29199.editorial 33103922

